# Electronic cleansing of tagged residue in CT colonography: what radiologists need to know

**DOI:** 10.1186/s13244-020-00848-9

**Published:** 2020-03-13

**Authors:** Thomas Mang, Christian Bräuer, Stefaan Gryspeerdt, Martina Scharitzer, Helmut Ringl, Philippe Lefere

**Affiliations:** 1 Department of Biomedical Imaging and Image-guided Therapy, Medical University of Vienna, Währinger Gürtel 18–20, A-1090 Vienna, Austria; 2 Department of Radiology, AZ Delta, Bruggesteenweg 90, B-8800 Roeselare, Belgium; 3 Department of Radiology, Danube Hospital Vienna, Langobardenstrasse 122, A-1220 Wien, Austria

**Keywords:** CT colonography, Virtual colonoscopy, Electronic cleansing, Faecal tagging, Colorectal polyps

## Abstract

CT colonography (CTC) is the radiological examination of choice for the diagnosis of colorectal neoplasia. Faecal tagging is considered a mandatory part of bowel preparation. However, the colonic mucosa, obscured by tagged residue, is not accessible to endoluminal 3D views and requires time-consuming 2D evaluation. Electronic cleansing (EC) software algorithms can overcome this limitation by digitally subtracting tagged residue from the colonic lumen. Ideally, this enables a seamless 3D endoluminal evaluation. Despite this benefit, EC is a potential source of a wide range of artefacts. Accurate EC requires proper CTC examination technique and faecal tagging. The digital subtraction process has been shown to affect the relevant morphological features of both colonic anatomy and colonic lesions, if submerged under faecal residue. This article summarises the potential effects of EC on CTC imaging, the consequences for reporting and patient management, and strategies to avoid pitfalls. Furthermore, potentially negative effects on clinical reporting and patient management are shown, and problem-solving techniques, as well as recommendations for the appropriate use of EC techniques, are presented. Radiologists using EC should be familiar with EC-related effects on polyp size and also with correct measurement techniques.

## Key points

- Electronic cleansing (EC) software digitally subtracts tagged residue from the colonic lumen to enable a seamless endoluminal colonic evaluation.
- EC has the potential to reduce reading times and improve polyp detection.
- EC can affect the morphologic appearance of colonic anatomy and pathology and is a potential source of artefacts.
- EC-related pitfalls can be avoided by evaluation of findings on the corresponding unsubtracted image data.

## Background

CT colonography (CTC) is recommended as the radiological examination of choice for the diagnosis of colorectal neoplasia [1]. It is indicated in patients with incomplete or contraindicated colonoscopy and serves as a diagnostic option to screen for colorectal cancer and adenomas [1–3]. It is based on a low-dose, thin-section CT scan of the cleansed and distended colon in both the supine and prone positions. For bowel preparation, patients undergo a combination of a low-fibre or clear-liquid diet, oral administration of laxatives, and faecal tagging the day before CTC. Colonic distension with complete visualisation of all colonic segments is achieved with insufflation of CO_2_ or air by a thin, flexible rectal catheter. Data evaluation is performed using dedicated CTC software, which enables the simultaneous evaluation of endoluminal 3D views and 2D multiplanar images of the colon. In addition to results from an American screening study [4], the importance of endoluminal 3D evaluations was highlighted in a study that evaluated the performance of CTC in the English Bowel Cancer Screening programme. However, 3D evaluation is known to be more time-consuming than 2D interpretation alone.

A thoroughly cleansed and well-distended colon is a prerequisite of a high-quality examination [5]. The evaluation of CTC image data can be negatively affected by residual stool and fluid in the colon, with faecal residue simulating and/or obscuring colonic lesions and residual fluid obscuring submerged lesions.

“Faecal tagging” significantly increases the CT attenuation of residual stool and fluid. This is achieved by oral administration of positive contrast media, such as iodine and/or barium, during the bowel preparation process [6]. Tagging agents are, therefore, administered to the patient usually the day before the examination or, at the latest, 3 h prior to the examination.

**Fig. 1:**
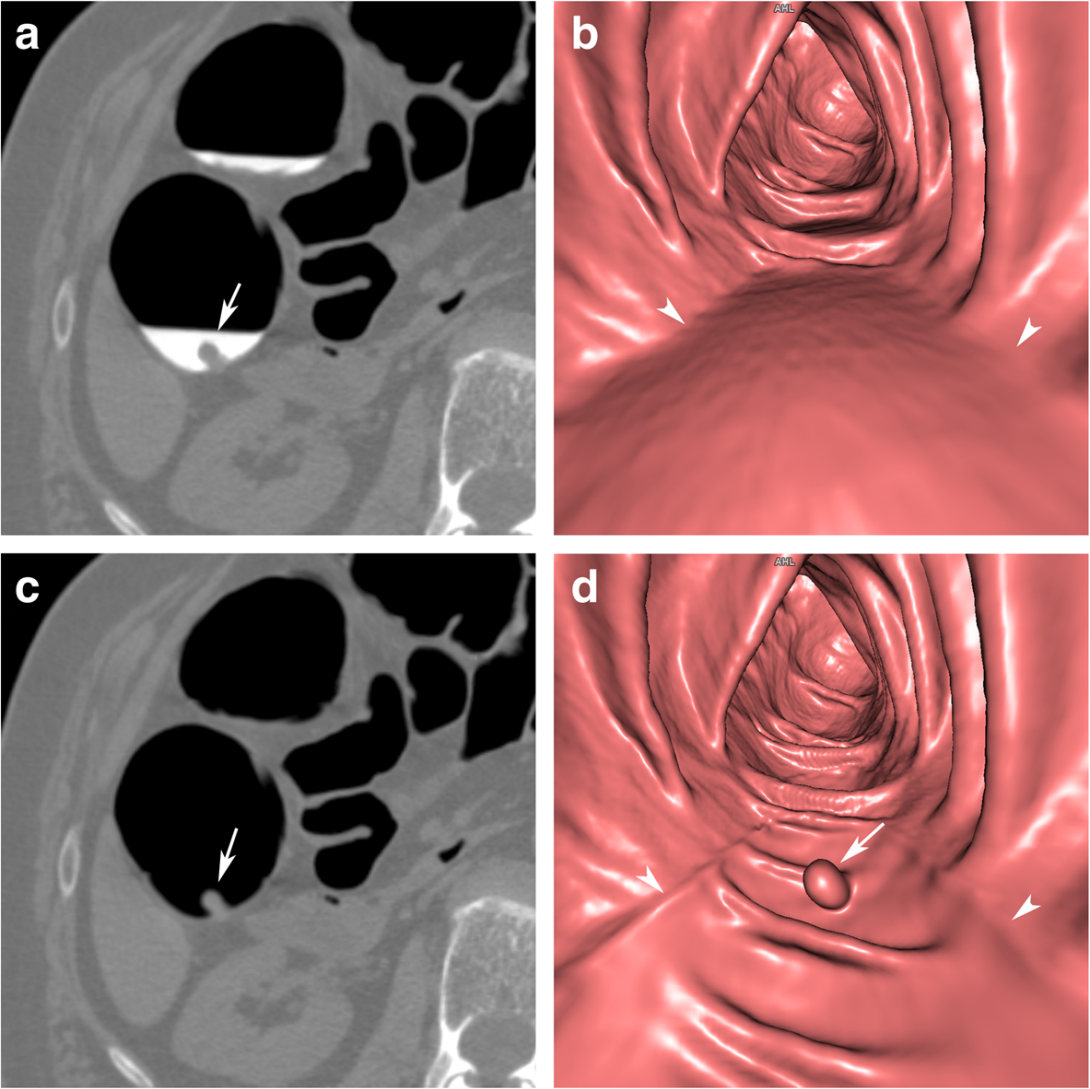
Sessile polyp in the ascending colon, submerged entirely under tagged residual fluid. **a** Axial 2D view shows a sessile polyp (arrow) with a homogeneous soft tissue attenuation within the tagged hyper-dense fluid. **b** In the corresponding endoluminal view, only a horizontal fluid layer is seen (arrowheads). **c** After EC, the tagged fluid is subtracted from the 2D view. **d** The polyp also becomes visible on endoluminal 3D views and presents with a round shape and a smooth surface (arrow). A linear artefact often occurs at the air-fluid interface (arrowheads). It helps to identify parts of the colon wall that were originally submerged. Note that the haustral folds are preserved

As a consequence, residual stool and fluid, tagged with contrast media, can easily be identified as faecal residue due to its high CT attenuation values (hyperdense), compared to intrinsic soft tissue lesions (soft tissue attenuation), and vice versa. Soft tissue lesions submerged within hyperdense tagged fluid can easily be depicted on 2D images as negative filling defects (Fig. 1a).

Faecal tagging was shown to increase the sensitivity, as well as the specificity, of CTC, and is considered a mandatory part of bowel preparation [5, 6]. In addition to faecal tagging, endoluminal 3D views were shown to improve polyp detection rates and were recommended to be incorporated into CTC evaluation strategies [5, 7]. However, even after faecal tagging, the colonic mucosa, obscured by tagged faecal residue, is not accessible to endoluminal 3D views (Fig. 1b). This requires repetitive interruptions of the 3D endoluminal fly-through for 2D evaluation for every submerged colonic area or segment. This is tedious and time-consuming.

### Electronic cleansing

Electronic cleansing (EC) is a software application, designed to overcome this limitation simply by digitally subtracting tagged residue from the CTC images. This subtraction process is based on the high attenuation values of tagged residue and includes the following steps. All voxels within the colonic lumen with a CT density value that exceeds a dedicated threshold value will be recognised by the EC algorithm as potentially tagged faecal residue. This threshold is pre-set within the applied electronic cleansing software and cannot be adjusted by the radiologist. Faecal residue, as well as intraluminal lesions with an attenuation lower than 100 HU, will not be recognised as tagged and will not be electronically removed. Tagged faecal residue with a CT attenuation above the threshold of 100 HU will then be digitally extracted from the datasets by attributing the density value of air to it [8].

Accordingly, the colonic wall, covered by faecal residue, becomes endoluminally visible in both 2D and 3D CTC images (Fig. 1c, d).

By enabling 3D evaluation of colonic mucosa that would otherwise be obscured, the entire colonic wall can be seamlessly evaluated on endoluminal 3D images when EC is employed [9]. Thus, the radiologist’s reading time is reduced because numerous time-consuming switches from 3D endoluminal to corresponding 2D views are eliminated in every single submerged segment [7, 10, 11].

Furthermore, the digital subtraction of intraluminal tagged faecal residue from CTC images may eliminate potential sources of false-negative and false-positive diagnoses and thereby improve polyp detection [10, 12, 13]. By digitally removing residual stool that could be mistaken for polyps from the colonic lumen, the specificity of data evaluation may be improved. Furthermore, polyps covered by tagged, solid residue or fluid can be depicted more easily by simply using endoluminal 3D images where polyps have been shown to be more conspicuous, compared to 2D planar views [4].

### Procedure details

In this pictorial review article, we report our observations of the clinical use of a commercially available EC algorithm (Tagged Stool Subtraction, Syngo CT Colonography VB10, Siemens Healthcare) that was applied on a database of colonoscopy-validated CTC screening-patient datasets [8], as well as in daily radiological practice. We describe the potential effects of EC on the appearance of the intraluminal anatomical morphology, as well as on the morphology and size of colonic lesions submerged under tagged residue. Furthermore, we demonstrate the potential negative effects on clinical reporting and patient management and describe problem-solving techniques, as well as recommendations for the appropriate use of EC techniques.

The observations about EC, presented within this review, are limited to the EC algorithm used by the authors. However, since the basic principle of EC might also be applied in other approaches, it cannot be excluded that artefacts and changes in lesion characteristics will also appear with other algorithms, as supported by observations in previous studies [11, 12, 14].

## Clinical findings

Ideally, after EC, only intraluminal, tagged, residual stool and fluid should have been digitally removed from the colon. Intrinsic colonic structures, such as the colonic wall, the semilunar folds, and tumoural lesions of the colon, should not be affected by EC, in segments with or without tagged faecal residue. Therefore, these structures must maintain their typical morphologic appearance when EC algorithms are applied.

### Normal colonic wall

In CTC examinations, with adequate colonic distension, the wall of the distended normal colon is very thin, measuring less than 2 mm [15]. Typically, it should be barely perceptible on 2D CTC images and may be better depicted with abdominal window settings. The normal colonic wall has a soft tissue attenuation on 2D images. It may show a slight enhancement after intravenous contrast media application. On endoluminal 3D images, the normal colon presents with a smooth surface.

Submerged segments are recognised as a horizontal fluid layer on endoluminal 3D views with a hyperdense attenuation on 2D planar views.

After digital subtraction, electronically cleansed colonic segments often present with a slightly smoother surface on endoluminal 3D views than segments without faecal residue. On 2D views, the colonic wall maintains its normal thickness. A linear artefact often occurs at the air-fluid interface, both on 2D and on endoluminal 3D views. This artefact may help radiologists to recognize colonic areas and segments that were electronically cleansed during the reading process (Fig. 1d).

### Colonic lesions

Intrinsic colonic lesions should have the same morphologic features before and after electronic cleansing [16]. The basic CTC imaging criteria, necessary to sufficiently characterise a detected colonic filling defect, are morphology, which is related to the shape and surface of a finding; the internal structure, which describes attenuation and homogeneity; and finally, mobility, which shows whether or not a finding is attached to the colonic wall.

Sessile polyps are hemispheric, round, or oval filling defects with a smooth or lobulated surface, with a base equal to or larger than the lesion’s height (Fig. 1). Colonic polyps arise from the colonic wall and are generally expected to maintain their intraluminal position when the patient is turned.

Pedunculated polyps typically present a round, oval, or lobulated head. The polyp head is connected to the colonic wall by a stalk. Therefore, the polyp head is mobile and will always follow gravity to the dependent colonic wall.

Non-polypoid, or so-called flat lesions, are characterised by their low height (< 3 mm) compared to their width. At CTC, they present as plaque-like elevations of the colonic wall, with a smooth or nodular surface.

Colorectal cancer occurs as a focal polypoid mass or a semi-circular or annular thickening of the colonic wall, typically with a short segmental extension (< 5 cm) and overhanging edges and shouldering at the transition to the adjacent normal mucosa.

On 2D views with abdominal window settings, colonic polyps, and cancers, demonstrate a homogeneous internal soft tissue attenuation.

## The importance of adequate bowel preparation and faecal tagging

It is important to always keep in mind that EC is only a digital manipulation of image datasets. Furthermore, its basic function relies inevitably on the quality of bowel preparation, and, more specifically, on the attenuation and homogeneity of faecal tagging. The proper function of EC requires a homogeneous tagging of faecal residue with a high CT attenuation that is above a minimum density threshold at which the software recognises faecal residue as tagged.

In examinations with insufficient bowel preparation and/or faecal tagging, the proper function of EC will be impaired [9, 14] and artefacts may appear in the image data. Therefore, subtracted image data should be interpreted with caution.

### Insufficient tagging

**Fig. 2:**
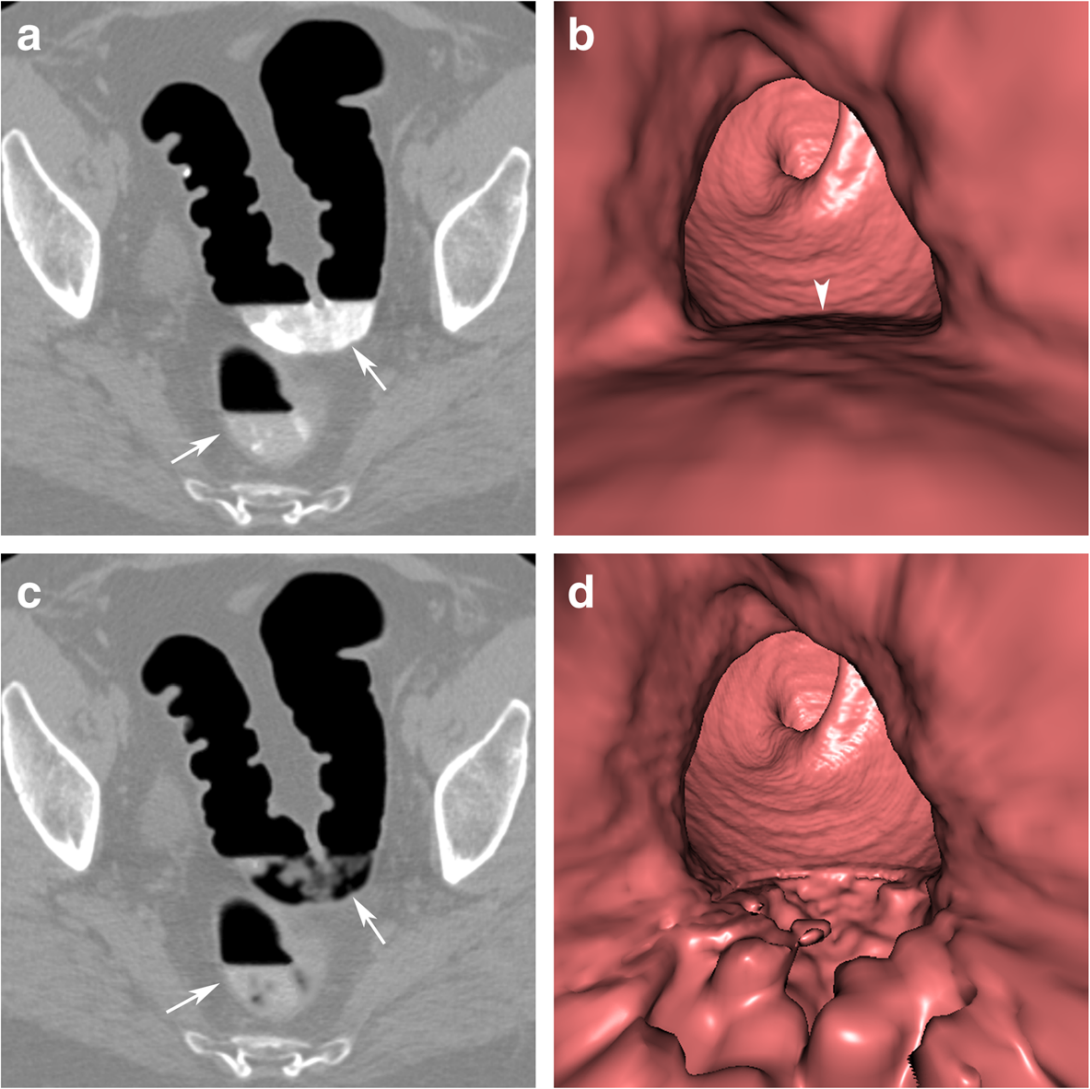
Incomplete EC due to inhomogeneously tagged faecal residue. **a** Faecal residues are not adequately tagged, resulting in an inhomogeneous appearance on axial 2D images (arrows). **b** A horizontal fluid layer is seen endoluminally before EC (arrowhead). **c** After EC, only sufficiently tagged parts of the residue can be subtracted, while the other parts remain in the colon lumen (arrow). **d** This leads to a bizarre, irregular luminal filling defect on 3D endoluminal views

If faecal tagging did not sufficiently increase the attenuation values of faecal residue to the minimum threshold of the EC algorithm (e.g., 100 HU or higher), it will not be recognised by the algorithm as tagged. This can be the result of inappropriate timing of the administration of tagging agents so that the contrast media either did not arrive in the colon or has already left the colon at the time of the examination. Depending on the tagging regimen used, tagging agents should be administered in the afternoon or evening before the examination if it is scheduled in the morning, but at least 3 h prior to the examination. In addition, undertagging may occur if the amount of administered contrast media is too small. This may occur in patients who are not compliant with the prescriptions of the preparation. Therefore, segments containing “undertagged” residue will not be digitally cleansed (Fig. 2).

### Inhomogeneous tagging/insufficient cleansing

If faecal residue contains indigestible food contents or untagged stool particles, it appears as inhomogeneous on 2D views after faecal tagging, with focal areas of high density and areas of low density. The computer algorithm, however, is able to subtract only hyperdense tagged parts of the residue, ignoring hypodense untagged particles. EC will then be incomplete with bizarre and irregular luminal filling defects on endoluminal 3D views. Unsubtracted planar 2D images are helpful to distinguish between incompletely subtracted faecal residue and colonic findings (Fig. 2).

### Solitary particles of untagged or poorly tagged stool

**Fig. 3:**
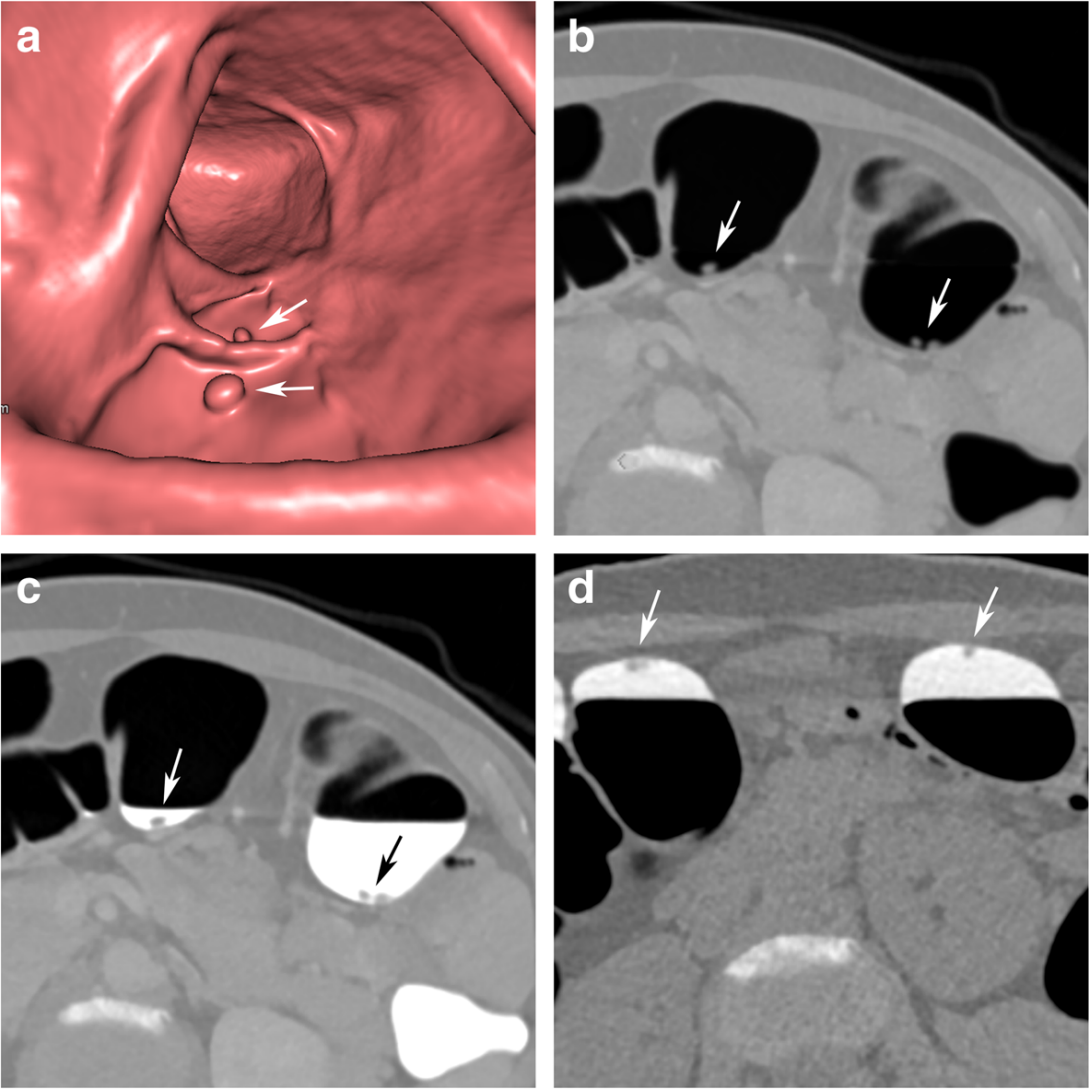
Pseudopolyps due to particles of untagged faecal residue in the transverse colon. **a** Endoluminal 3D and (**b**) axial 2D view after EC, showing multiple small sessile polypoid filling defects (arrows). On corresponding unsubtracted (**c**) prone and (**d**) supine 2D views, the filling defects shift with the residual fluid from the dorsal to the anterior section of the transverse colon (arrows). They are not connected to the colonic wall. This is a typical imaging feature of faecal residue

Solitary particles of untagged or poorly tagged stool, surrounded by tagged fluid, remain in the colonic lumen while tagged faecal residue is subtracted. Untagged stool may thereby simulate colonic polyps on endoluminal 3D views after EC and eventually cause a false-positive finding. However, in contrast to true colonic polyps, the residue often presents with an air inclusion or floats in a pool of contrast and is, therefore, not attached to the colonic wall. Consequently, it will show a positional shift to the dependent sections of the colonic segment between the prone and the supine CT acquisitions (Fig. 3) [17].

### Persistent polypoid deposits of tagged stool

**Fig. 4:**
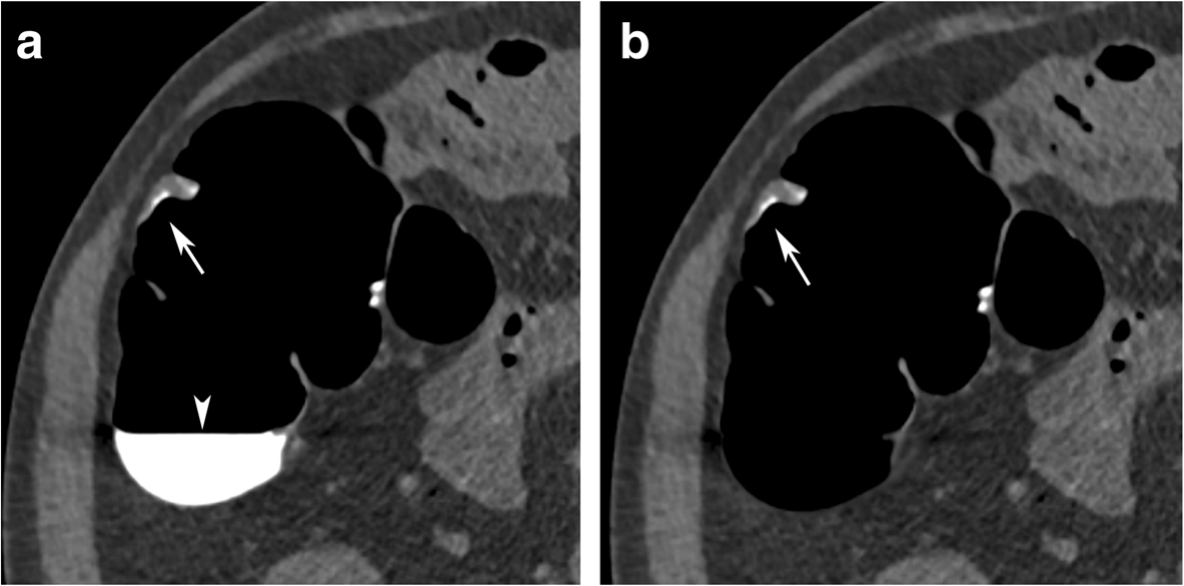
Persistent tagged residue on a flat lesion in the ascending colon. **a** On the axial 2D image before EC, the soft tissue plaque-like lesion is coated by a thin layer of hyperdense tagged residue (arrow). Tagged residual fluid is seen on the dependent colonic wall (arrowhead). **b** After EC, the layer of contrast media remains on the flat lesion (arrow) while the residual fluid was removed. Note that “contrast coating” of polyps has been reported to aid in polyp detection. Focal deposits of contrast media are therefore intentionally not removed by EC

Small focal deposits of tagged stool may not be subtracted by the algorithm. This is intended so that small or flat lesions that are coated with tagged material are not missed. This phenomenon, which has been described as being present in up to 79% of all flat polyps, may indicate the presence of serrated lesions [18, 19]. Persistent polypoid deposits of tagged stool are easily recognised as pseudolesions by their high density on 2D views (Fig 4).

It is, therefore, important to evaluate the corresponding 2D views to assess the attenuation characteristics of each filling defect and to identify soft tissue lesions that are covered by a layer of tagged residue.

## Artefacts related to the digital subtraction process

EC can be the source of a wide range of image artefacts that can affect the intraluminal aspect of the colon. Some of these artefacts have the potential to decrease the appearance and conspicuity of colonic lesions and others may simulate the presence of colonic lesions.

### Linear artefacts

After EC of tagged fluid, a linear artefact is often present at the air-fluid interface. It can be recognised on both 3D and 2D images and should not be confused with a true colonic structure or lesion. Radiologists who evaluate 3D endoluminal images after EC can use this artefact as a marker to identify submerged sections of the colon that were electronically cleansed. This can be helpful for the evaluation by taking special care in the analysis of findings located in electronically cleansed segments (Fig. 1d). In case of doubt, subtracted and unsubtracted images need to be compared.

### Pseudopolyps due to partial volume effects

Due to the image characteristics of CT, there is always a partial volume effect at the interface between the colonic wall, intraluminal air, and tagged residue. This may increase the attenuation values, specifically of those sections of the colon wall and air that are adjacent to hyperdense tagged residue. This may have a negative effect on the subtraction algorithms, leading to artefacts that simulate polypoid lesions, specifically if located close to semilunar folds.

**Fig. 5:**
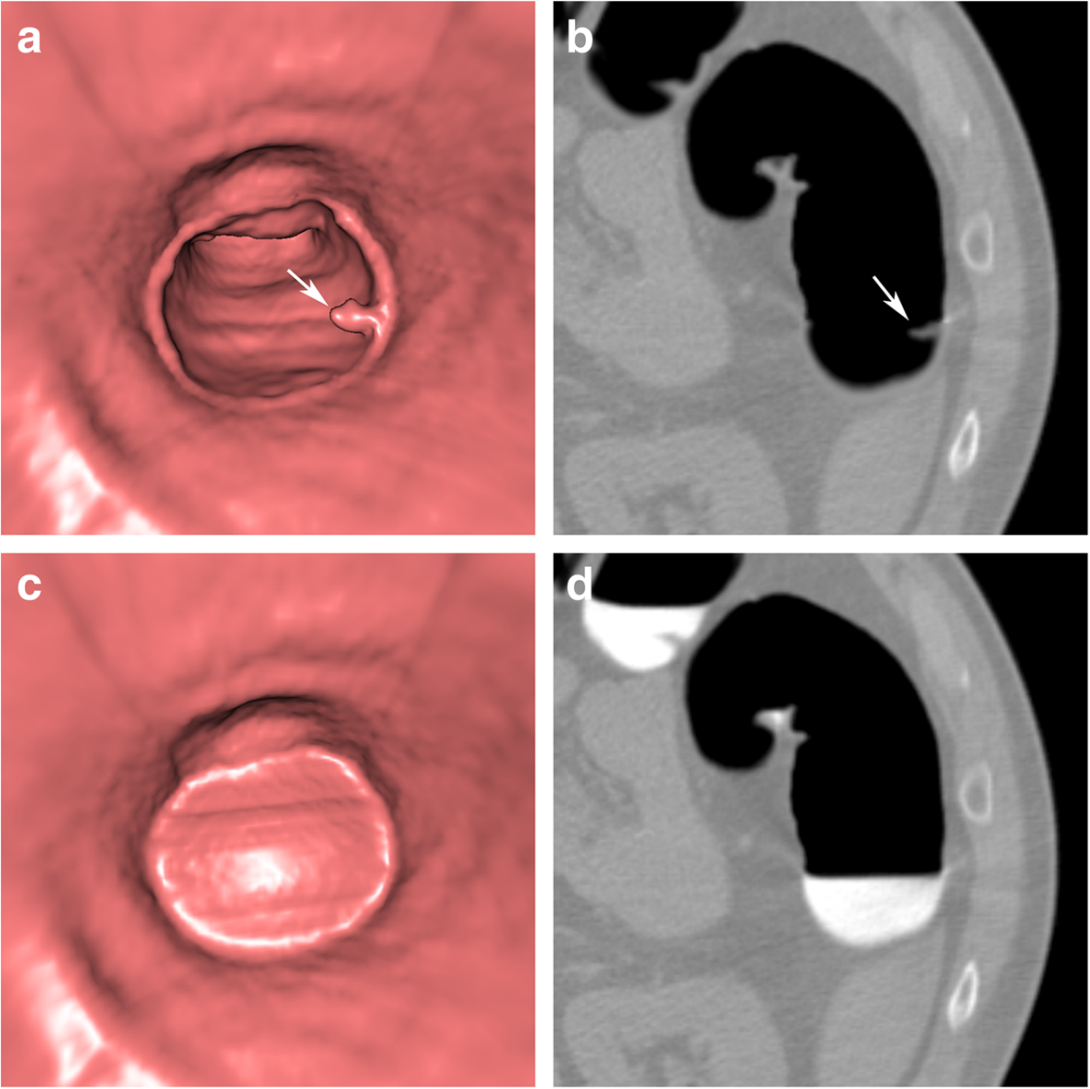
A pseudopolyp at the interface between tagged residue and air in the descending colon. **a** Endoluminal 3D and (**b**) axial 2D view after EC, showing irregular pseudopolypoid filling defects (arrow), visible at the interface between tagged residues and air. On corresponding unsubtracted (**c**) 3D and (**d**) 2D views, no lesion is seen

Pseudopolyps, related to partial volume effects, are always located at the interface between tagged residue and air. They typically present with a more angular bizarre morphology on endoluminal 3D and planar 2D views, atypically for true polypoid lesions. The corresponding unsubtracted 2D images are diagnostic and will not show any corresponding polypoid lesion (Fig. 5). Furthermore, there will be no corresponding findings in the other scanning position.

### Artefacts due to motion of tagged residual fluid

Intraluminal motion of tagged residual fluid during the CTC scans leads to artefacts at the interface between tagged residue and air. Ideally, the surface of a fluid level presents as a linear horizontal smooth layer. Movement of the patient, bowel peristalsis, but also fluid shifts from upper to lower segments will lead to motion of the intraluminal fluid with wave-like, bubble-like, or even an irregular appearance of the surface level on unsubtracted CTC images [20].

**Fig. 6:**
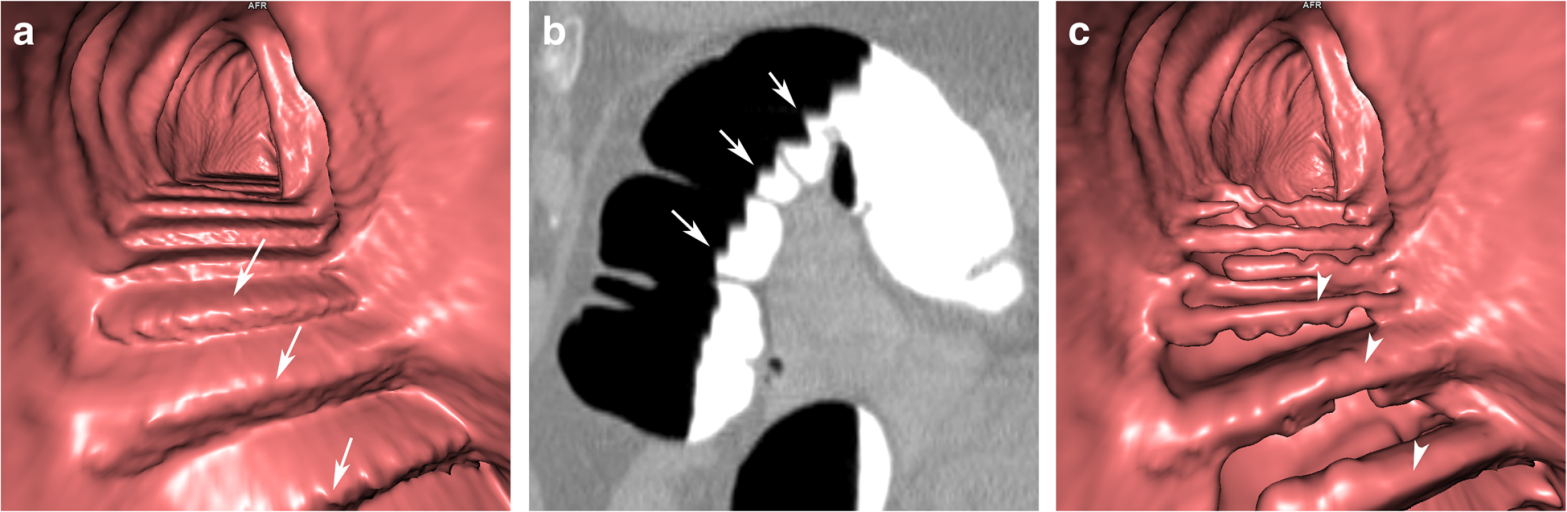
Fluid motion artefacts at the interface between tagged residues and air in the descending colon. **a** Endoluminal 3D view and (**b**) sagittal 2D view showing stair-step artefacts at the air-fluid layer related to fluid motion in the colonic lumen (arrows). Note that the colonic wall shows no artefacts. **c** EC is incomplete leading to a grid-like artefact at the air-fluid layer (arrowheads)

Fluid motion artefacts can present with either a wave-like or stair-step appearance at the fluid level or even as bizarre intraluminal structures on planar 2D and endoluminal 3D images. The colonic wall shows no related artefacts. The motion of intraluminal fluid impairs the subtraction process, which leads to electronic cleansing artefacts. After EC, grid-like or bizarre artefacts remain at the air-fluid interface (Fig. 6).

### Pseudopolyps due to trapped gas bubbles

**Fig. 7:**
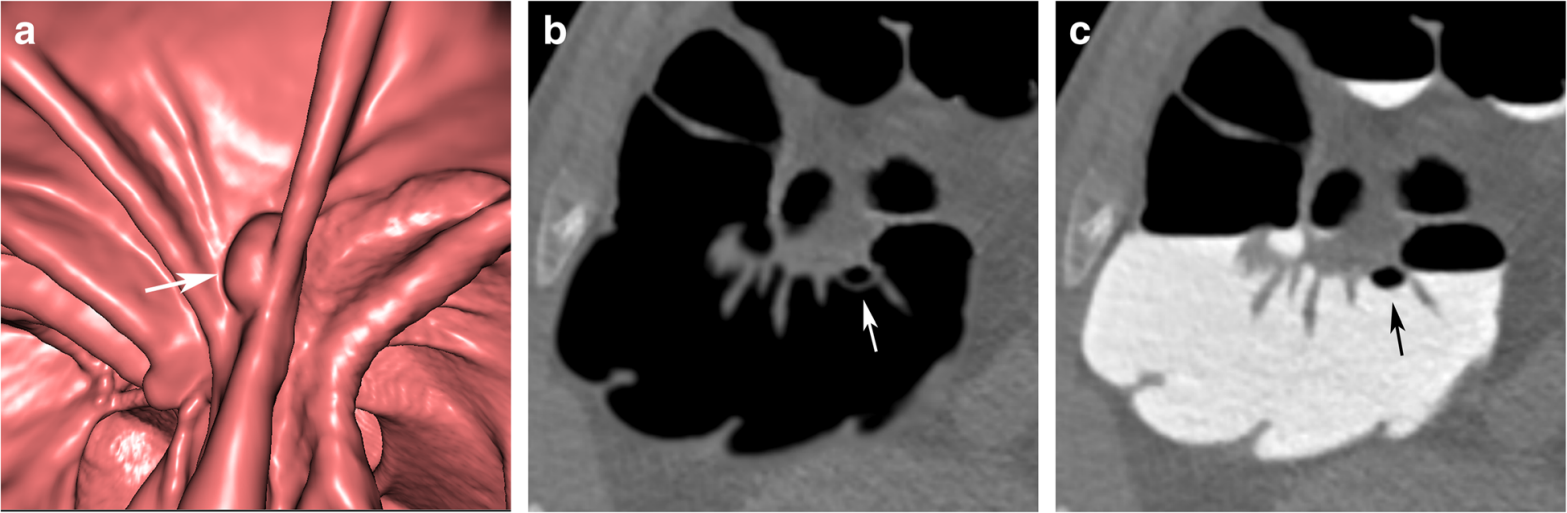
Pseudopolyp at the right colonic flexure due to trapped gas bubbles after EC. **a** In an endoluminal 3D view after EC, an oval filling defect is visible, simulating a sessile polyp (arrow). **b** In the corresponding 2D image after EC, a gas-filled cyst with a thin wall is seen (arrow). **c** In the unsubtracted 2D image, the round lesion correlates with a gas bubble trapped between two semilunar folds (arrow)

Small bubbles of gas, trapped in colonic segments that are completely filled with tagged residual fluid, may simulate sessile polypoid lesions after EC. This can happen if the surface layer of the gas bubble is not subtracted and remains within the colonic segment. On endoluminal 3D views, they present as sessile, round, polypoid findings with a smooth surface. On corresponding electronically cleansed 2D images, a gas-filled cystic finding with a thin wall is seen (Fig. 7). Since gas bubbles ascend within fluid, gas bubbles are always located on the upper parts of submerged segments but may also be trapped within deep haustra. They will, therefore, disappear in the other scanning position. Unsubtracted 2D views will identify the gas bubble.

## Artefacts on anatomic structures and colonic lesions

Artefacts, associated with EC, may not only simulate colonic lesions. They can also cause distortion of anatomic structures and of colonic lesions and thereby impair their proper radiologic evaluation.

### Semilunar folds

The semilunar folds are thin, crescent-shaped structures with a smooth surface and a soft tissue density. In unsubtracted 2D views, they are better recognised with wide window settings.

**Fig. 8:**
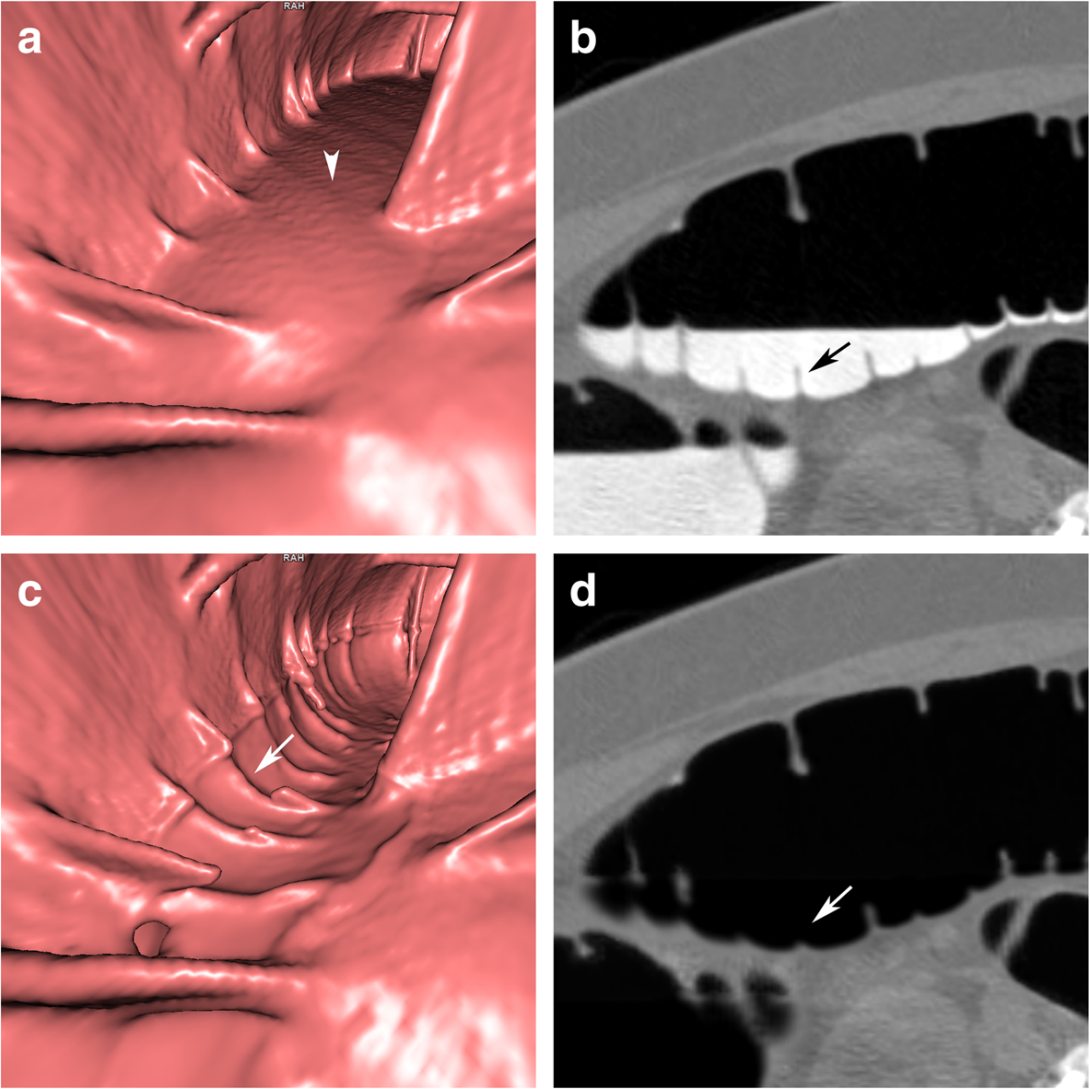
Pseudodefects of haustral folds, submerged under tagged residual fluid after EC. **a** On endoluminal 3D views, a horizontal fluid layer is found in the transverse colon, obscuring parts of the colonic wall before EC (arrowhead). Only those parts of the colon wall and haustral folds that lie above the fluid layer are visible. **b** On sagittal 2D views, thin haustral folds are submerged under tagged residual fluid (arrow). **c**, **d** After EC, the residual fluid is removed. Note that the submerged parts of haustral folds show defects, disappearing entirely (arrow), while those parts of the haustral folds that are not submerged do not change in appearance on (**c**) endoluminal 3D views and (**d**) sagittal 2D views

After EC, thin, semilunar folds, covered by tagged residue, may be either distorted or may disappear entirely together with the residue (Fig. 8). This can be caused by erroneous subtraction, resulting from pseudoenhancement of thin, semilunar folds that are surrounded on both sides by hyperdense tagged residues.

### Colonic wall

Similar to the semilunar folds, the colonic wall can be affected by the same mechanism. If two adjacent colonic segments, filled with tagged residue, are in direct contact, the colonic wall can also appear distorted with apparent colonic wall defects. Evaluation of unsubtracted image data is helpful to verify that the colonic wall is intact.

### Polyp morphology

**Fig. 9:**
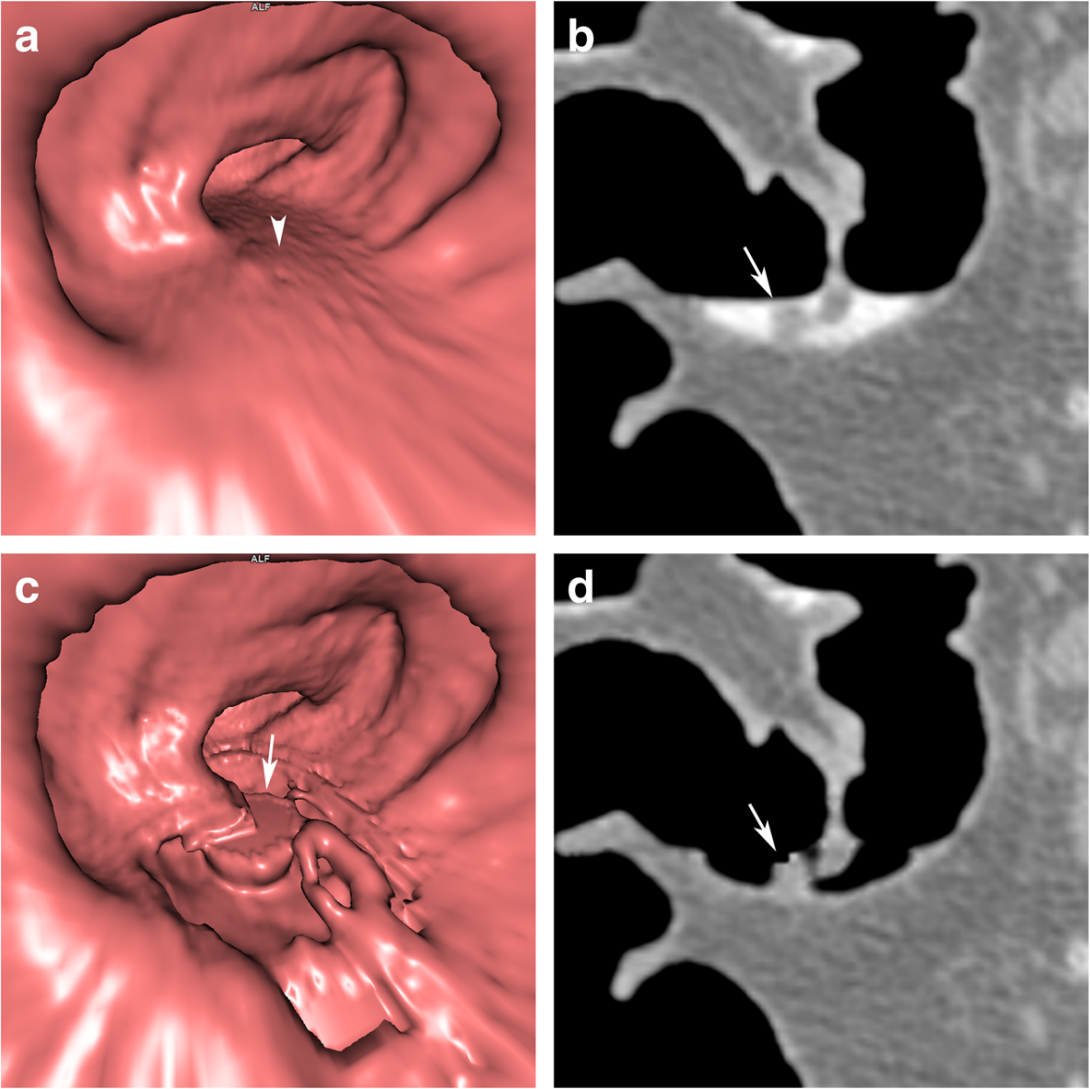
Changes in the morphologic appearance of a pedunculated colonic polyp in the sigmoid colon due to EC. **a** On endoluminal 3D views, a horizontal fluid layer in the descending colon obscures a pedunculated polyp before EC (arrowhead). **b** On the axial 2D image before EC, the soft tissue lesion is almost completely submerged in hyperdense tagged residues (arrow). **c** After EC, the polyp head presents with an irregular shape, with a reduced conspicuity on endoluminal 3D views (arrow). **d** Axial 2D image after EC, showing the soft tissue lesion associated with some linear artefacts (arrow). Note that unsubtracted 2D views are helpful to evaluate this finding

The correct assessment of a lesion’s morphology on CTC images is crucial to correctly classify a polypoid finding according to existing guidelines [21], e.g., to determine whether a polyp shows a pedunculated or sessile morphology, it is important to assess its connection to the colon wall. Depending on how much of the polyp is submerged in tagged residue and whether there is a layer of tagged fluid between the polyp head and the colon wall, the typical appearance of the polyp can be significantly affected by the subtraction process. Artefacts due to EC can make it more difficult to distinguish a pedunculated from a sessile polyp, e.g., if the polyp’s stalk is rendered incompletely or if the shape of the polyp is distorted due to changes on the lesion’s surface (Fig. 9).

### Polyp size

**Fig. 10:**
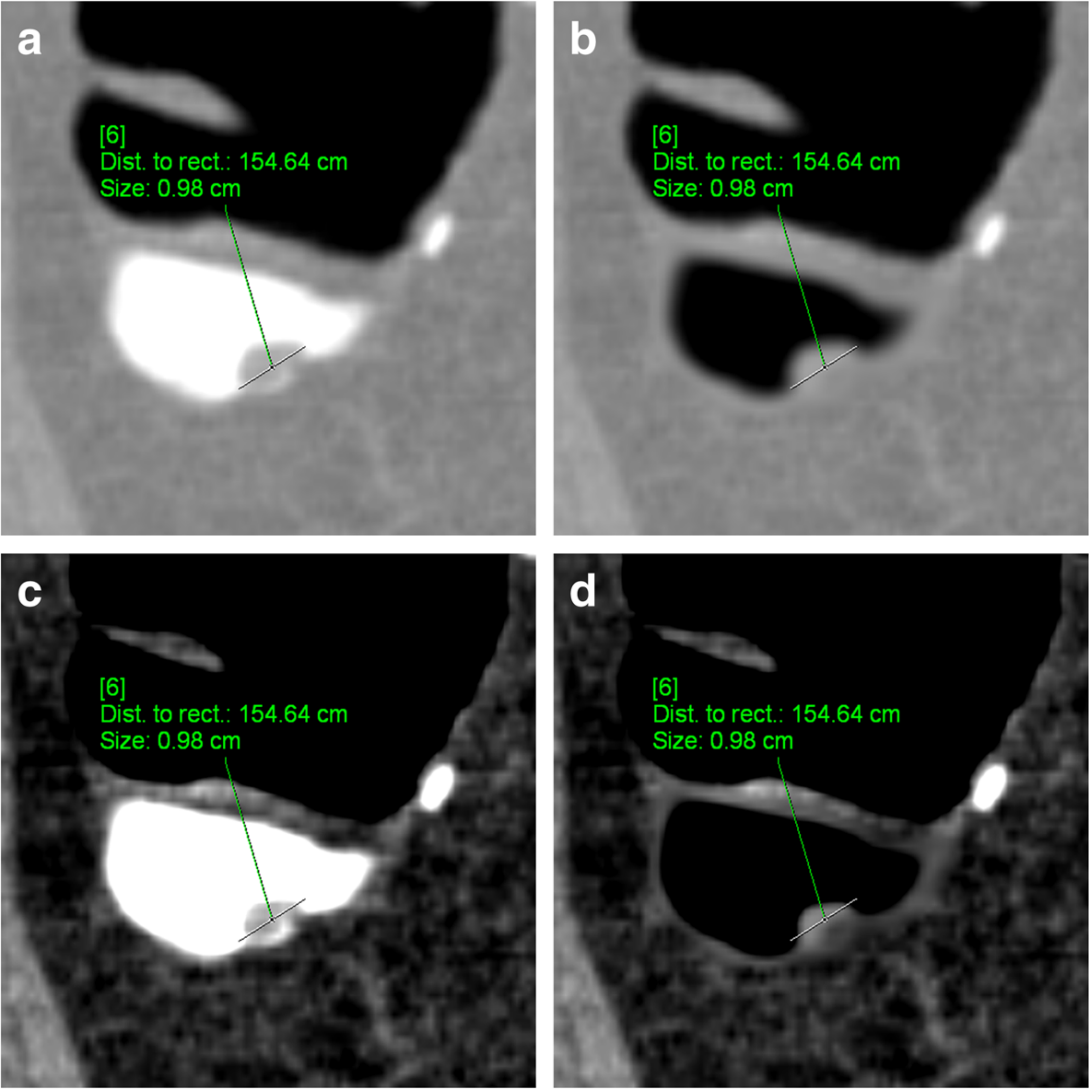
Apparent size reduction of a 10-mm submerged pedunculated polyp, located in the ascending colon, due to EC. **a** The size of the polyp is largest in the unsubtracted colon window (9.8 mm). **b** After EC, the polyp’s size decreased to 9.5 mm. **c**, **d** In the soft tissue window, the polyp size decreased from 7.0 mm before EC to 6.4 mm after EC.

It was recently observed that EC of tagged residue can lead to a significant reduction of the size of polyps submerged by tagged residue [22, 23]. Based on our experience, the size of polyps ≥ 6 mm decreased by 4.1% when measured in a colon CT window (W 1500/L − 150) and by 13.4% when measured in an abdominal one (W 400/L 40) (Fig. 10) [8]. A possible explanation for the reduction of the polyp size due to EC is the partial volume effect, which results in an attenuation gradient at the contrast-polyp interface. When using EC, the attenuation gradient of tagged residue to the colonic lesion must be transformed to a gradient of air to the colonic lesion. Currently, the new attenuation gradient/profile at the air-polyp interface shows a flatter profile than the one before EC, therefore resulting in a smaller appearance of the polyps.

### Importance of the polyp size

Adenomatous polyps are potential precursor lesions of colorectal cancer and are therefore a target lesion of CTC. The 10-year risk for colorectal cancer increases with polyp size. While it is 0.08% for diminutive polyps < 6 mm, it increases to 0.7% and 15.7% for small (6–9 mm) and large polyps (≥ 10 mm), respectively [24]. Therefore, in CTC, size is critical for the estimation of the clinical relevance of a polyp and for the therapeutic approach [5, 21]. While diminutive lesions < 6 mm may be ignored, there is a general consensus that all polyps ≥ 6 mm must be reported. With regard to treatment, large polyps ≥ 10 mm require endoscopic resection, while the management of small polyps between 6 and 10 mm has been discussed controversially. While European guidelines request colonoscopic resection [5], there is increasing evidence from longitudinal polyp studies that a less invasive approach that includes only follow-up should be supported [21].

Taking these considerations into account, knowledge of the apparent reduction of the polyp size is of crucial importance in determining the correct therapeutic approach. EC-related apparent size reductions of polyps can be the source of misclassification of lesions into the incorrect and smaller size category, and, therefore, lead to underestimation of lesions of potential clinical relevance.

**Fig. 11:**
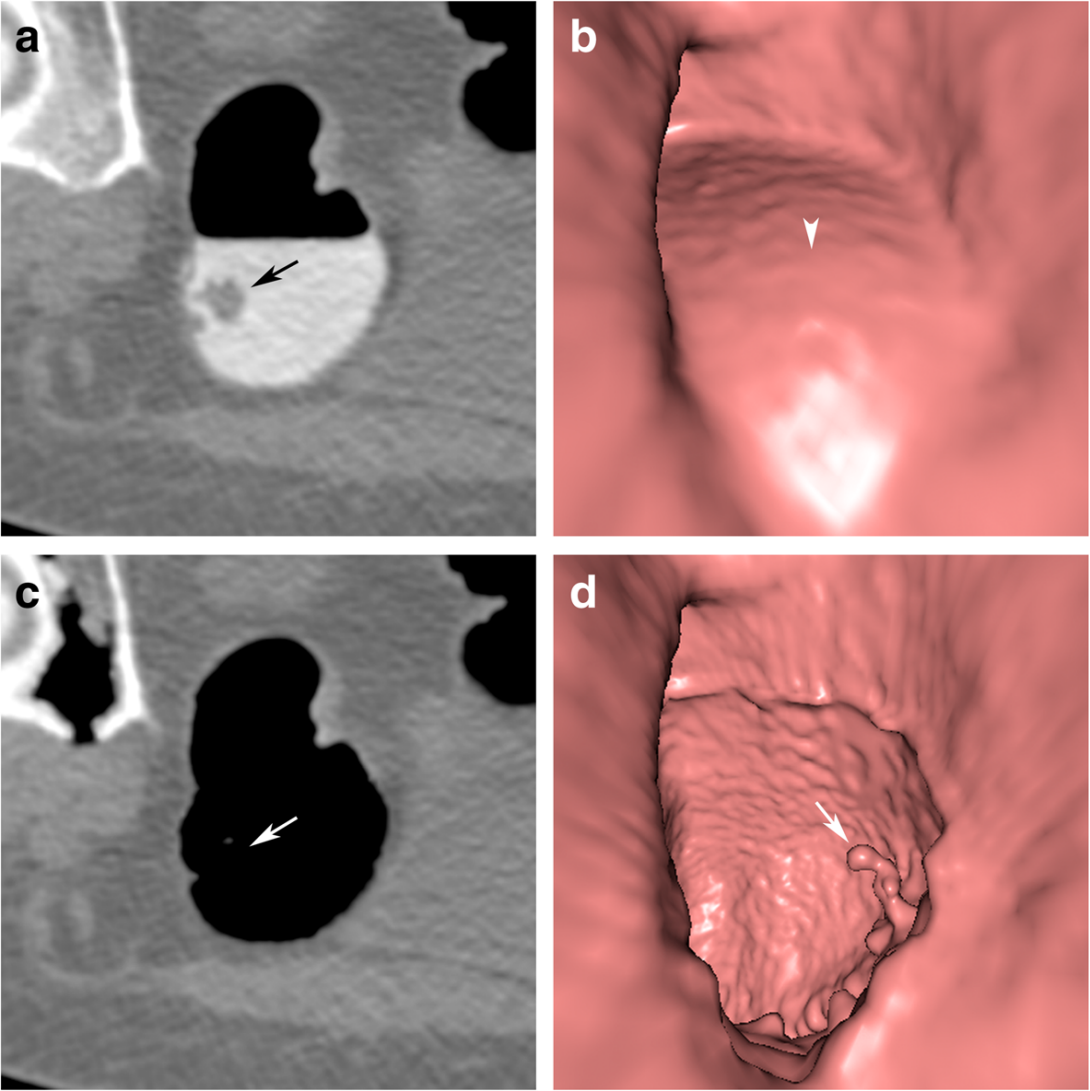
Apparent removal of a 9-mm submerged pedunculated polyp, located at the sigmoid colon, due to EC. **a** A 9-mm pedunculated polyp, submerged under tagged residual fluid, is clearly visible in unsubtracted 2D image data in a wide window (arrow). **b** Before EC, a horizontal fluid layer (arrowhead) is seen on unsubtracted ebdoluminal 3D views, obscuring parts of the colonic wall. **c** After EC, the polyp apparently disappears on the 2D images (arrow). **d** On endoluminal 3D images, the polyp presents as an irregular, distorted filling defect (arrow)

In the most unfavourable case, this EC-related size reduction could cause polyps to entirely disappear together with tagged residue (Fig. 11). This effect is specifically observed in narrow CT window settings.

EC-related measurement errors can be avoided by measuring the polyp size on unsubtracted CT image data, using a wide window setting. This ensures the most accurate reporting of polyp size and, therefore, also the appropriate therapeutic procedure for the patient.

## Conclusion

Computer algorithms for electronic cleansing of tagged faecal residue from the colonic lumen have the potential to improve colonic evaluation, first, by allowing a seamless and time-efficient 3D endoluminal evaluation of colonic segments filled with tagged residual fluid, and second, by improving the conspicuity of lesions that are submerged by tagged faecal residue.

However, the potential usefulness of this software application is still limited by a number of artefacts that can affect the colonic evaluation and interpretation, as well as the size measurement of colonic lesions that are submerged under tagged faecal residue. Future strategies to further reduce EC-related pitfalls and artefacts may include EC schemes that are based on the material decomposition capability of dual-energy CT [25, 26].

Radiologists, when incorporating EC algorithms into their routine workflow, need to be aware of the potentially adverse effects associated with the subtraction process. Suspicious colonic findings that are encountered on digitally subtracted image data need to be correlated with the corresponding unsubtracted views to prevent EC-related pitfalls. To avoid underestimation of the polyp size, measurements of submerged polyps should be performed on unsubtracted image data in a colon window setting.

## Data Availability

Not applicable

## Abbreviations

2D: Two-dimensional
3D: Three-dimensional
CT: Computed tomography
CTC: CT colonography
EC: Electronic cleansing
